# Severe complications of intrauterine device migration: Case reports of rectal perforation and omental localization

**DOI:** 10.1016/j.radcr.2024.07.073

**Published:** 2024-08-05

**Authors:** Hanae Benchaou, Marouane Boukroute, Ayoub Aouragh, Youssef El Aloua, Abdelmajide Regragui, Ibtissam Bellajdel, Zaineb chetbi, Hafsa Taheri, Hanane Saadi, Ahmed Mimouni

**Affiliations:** a Department of Obstetrics and Gynecology, Mohammed VI University Hospital Center, Oujda, Morocco; b Faculty of Medicine and Pharmacy, Mohammed First University Oujda, oriental state, Morocco

**Keywords:** Intrauterine device, Cyclical rectal bleeding, Hysteroscopy, Rectoscopy, Laparoscopy

## Abstract

Migration of intrauterine devices (IUD) is uncommon; however, it can lead to increasingly severe complications, ranging from simple pelvic pain to potentially lethal septic shock. Therefore, diagnostic and therapeutic approaches combining endoscopic paraclinical examinations with surgical intervention need further investigation by clinicians to ensure better management. This study presents 2 rare cases where a migrating intrauterine device at the rectal level was removed via hysteroscopic approach and a second one was removed via laparoscopy.

## Introduction

Intrauterine devices (IUDs) are effective long-term contraception methods, offering women flexibility in terms of duration of use. However, these devices can present septic complications, and more rarely uterine perforation, estimated at 0.5 to 1 per 1,000 insertions [1]. Consequently, a misdiagnosis can progress to peritonitis, and in extreme cases, to septic shock, which can be fatal. The described locations of migrating IUDs, once the uterine wall is breached, are extremely diverse. Thus, migrated IUDs have been found in all quadrants of the abdomen, particularly in the Douglas pouch, broad ligament, and omentum. Migration to the rectal region remains a rare occurrence, presenting specific diagnostic and therapeutic challenges [2].

## Case presentation 1

Ms. H.S, a 26-year-old, G2P2, followed for hypothyroidism with Levothyroxine, presented with cyclic rectal bleeding of low abundance lasting for 6 months, 2 months after insertion of a copper IUD. Clinical examination revealed a conscious patient stable neurologically, respiratorily, and hemodynamically.

Gynecological examination showed a soft abdomen without tenderness or guarding. Vaginal examination followed by a speculum exam revealed a normal-sized uterus with slight tenderness on uterine mobilization, a normal-looking cervix, healthy vaginal fornices, no bleeding, and absence of IUD string visualization.

**Fig. 1:**
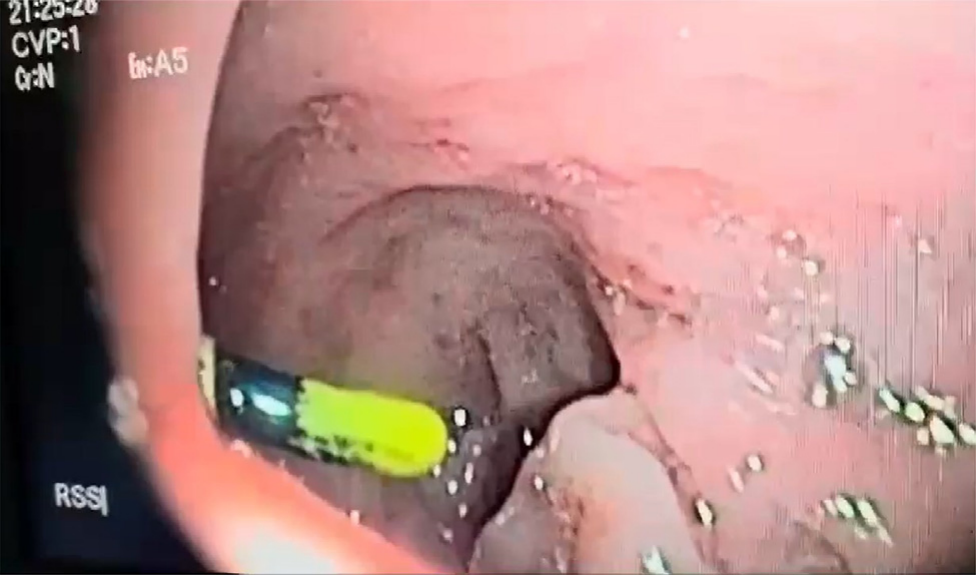
Colorectosigmoidoscopy revealing a foreign body resembling an intrauterine device located 15 cm from the anal margin.

Rectal examination was unremarkable, prompting rectosigmoidoscopy, which revealed a foreign body 15 cm from the anal margin (Fig. 1).

**Fig. 2:**
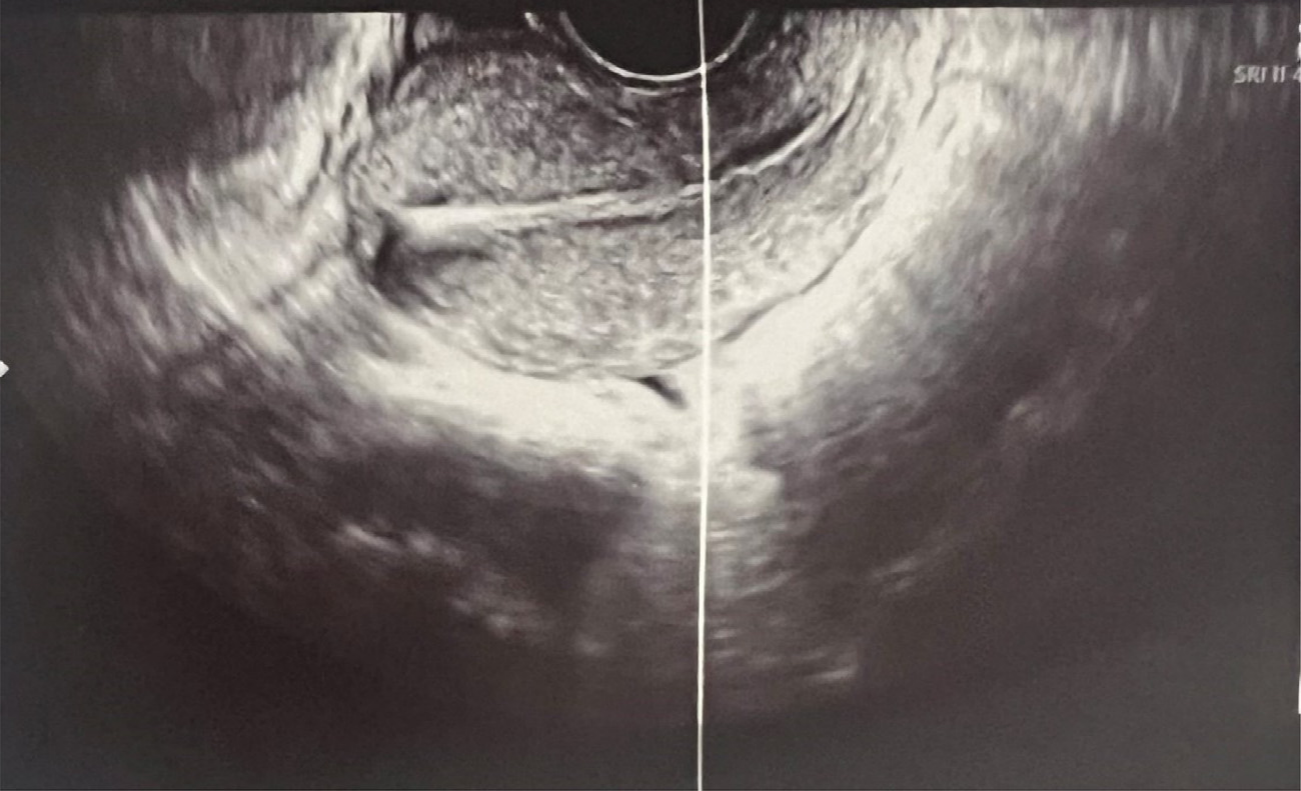
A pelvic ultrasound revealed a normal-sized uterus measuring 66*37 mm with homogeneous echostructure and regular contour. An intrauterine device (IUD) was observed intra uterine, positioned 17 mm from the uterine fundus.

Pelvic ultrasound showed a normal-sized uterus measuring 66*37 mm with homogeneous echostructure and regular contour, and an intrauterine IUD 17mm from the uterine fundus (Fig. 2).

**Fig. 3:**
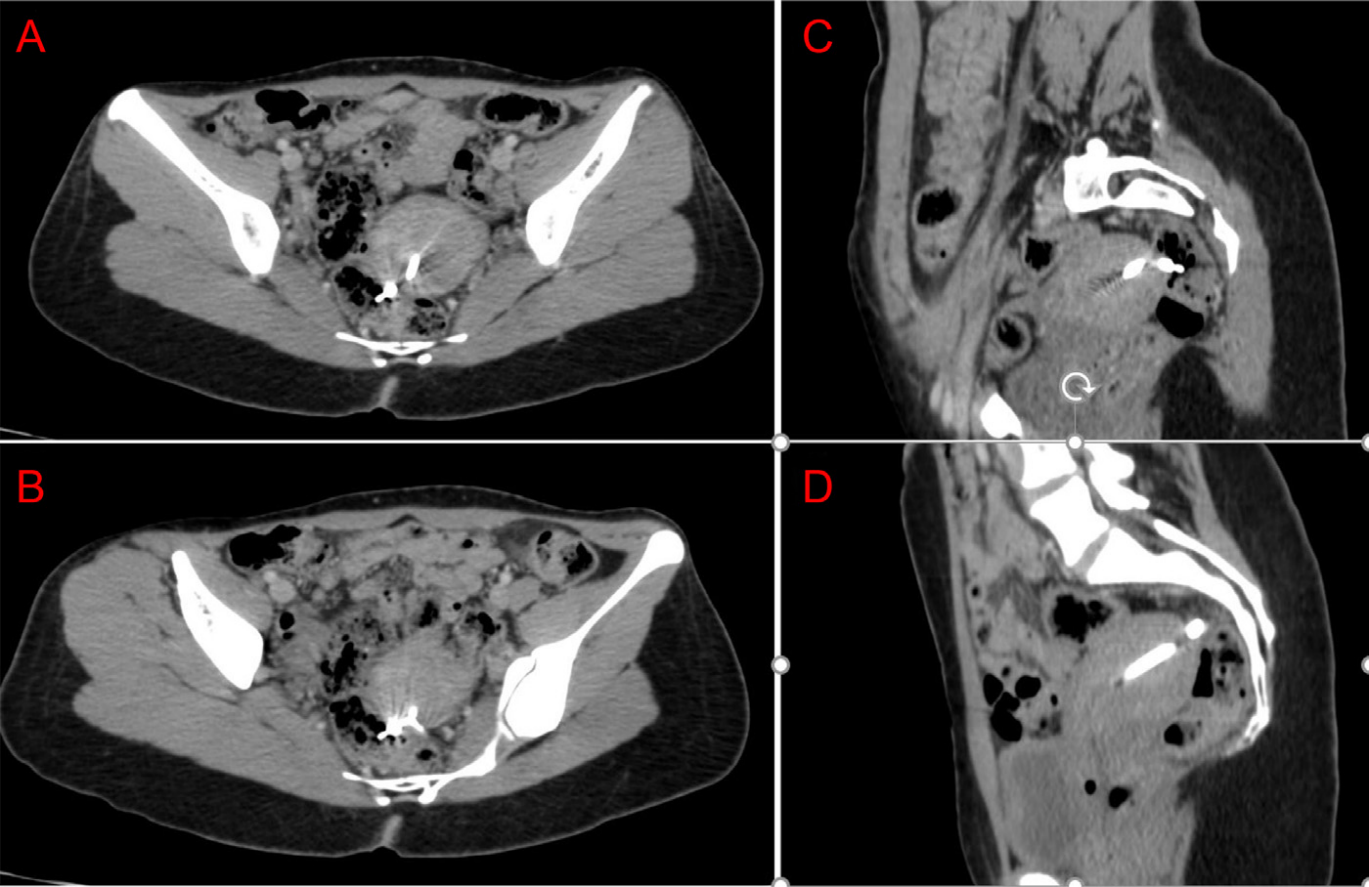
Axial (A,B) and sagittal (C,D) CT, showing an intrauterine device, which has migrated from the lumen of the uterus through the uterine wall and part of the device is now intra-rectally located.

Consequently, an abdominopelvic CT scan was performed to determine the exact location, showing the IUD positioned at the uterine fundus perforating the postero-lateral uterine wall and the upper rectum, 14 cm from the anal margin, with partial migration of the IUD into the rectum, without signs of pelvic or abdominal fluid collection (Fig. 3).

The rectal perforation orifice was very small, as shown by the colonoscopic images. To avoid enlarging the orifice surface, the decision was made to proceed with hysteroscopic removal despite the infectious risk. However, the abdominal cavity was not contaminated, as indicated by the CRP level and the patient's clinical condition (no abdominal pain, no fever spikes). Therefore, hysteroscopy under spinal anesthesia was performed, allowing visualization of the string, the copper-covered base, and the 2 branches, with the left branch penetrating the posterior wall. The migrating IUD was removed without resistance under hysteroscopy.

The follow-up involved the administration of triple preventive antibiotic therapy, including an aminoglycoside, a third-generation cephalosporin, and an imidazole. No additional procedures were performed, and a 1-month postoperative follow-up showed uneventful recovery without signs of genitodigestive fistula.

## Case presentation 2

A 30-year-old female patient, gravida 4 para 3, with 3 children delivered vaginally and no notable medical history, was referred from a health center due to a suspected uterine perforation during the insertion of an intrauterine device (IUD) 2 days prior. The patient reported acute pelvic pain during the IUD placement.

Clinical examination revealed an afebrile, conscious patient, hemodynamically stable with a heart rate of 84 bpm, blood pressure of 124/70 mmHg, and stable respiratory status with an oxygen saturation of 97%. Abdominal examination showed a soft abdomen with slight tenderness around the periumbilical area.

Gynecological examination did not reveal any discharge or bleeding, and the IUD string was not visible. There was pelvic pain upon bimanual palpation of the uterus and manipulation of the cervix. Vaginal examination revealed a long, closed posterior cervix with a clean withdrawal of the examining finger. Speculum examination showed healthy vaginal walls with no visible IUD string.

An abdominal X-ray indicated the presence of the IUD in the abdominal cavity outside the uterus. Given the suspicion of uterine perforation with migration of the IUD, a laparoscopy was performed to remove the IUD and assess for any involvement of neighboring organs.

**Fig. 4:**
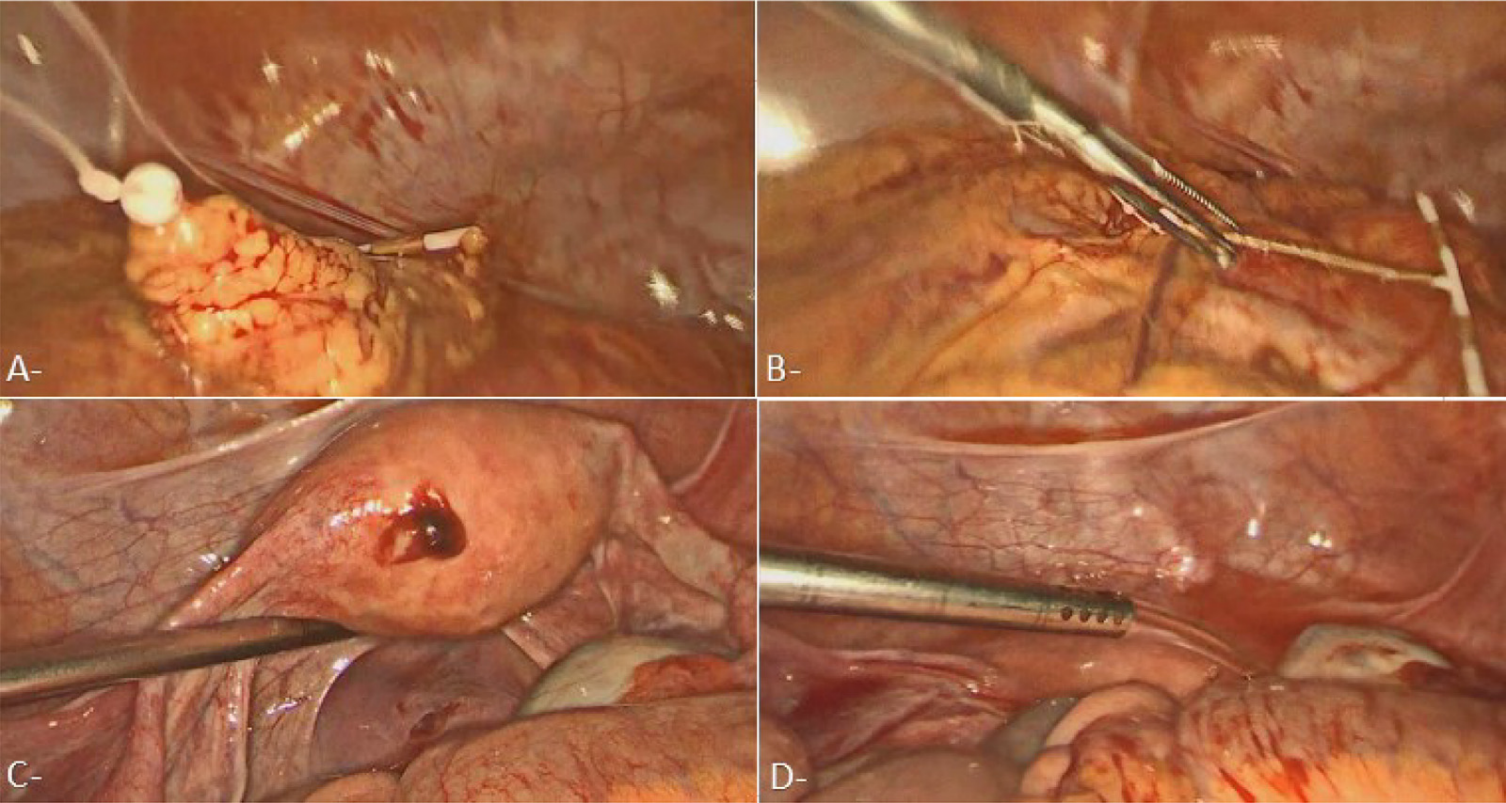
operating images during exploratory laparoscopy showing (A) The intrauterine device found within the peritoneal cavity, embedded in the omentum. (B) Gentle extraction of the intrauterine device was performed using forceps. (C) Identified uterine fundal perforation site. (D) The peritoneal cavity irrigated with saline solution.

The laparoscopy (Fig. 4) involved a systematic visual inspection of the abdominal cavity and pelvic organs to assess the extent of the IUD migration. The IUD was located within the omentum. The omentum was mobilized and the IUD was carefully disengaged, followed by its gentle extraction using laparoscopic forceps. The neighboring structures, including the intestines, bladder, and vascular networks, were evaluated. Hemostasis was verified in the area of IUD removal, and the peritoneal cavity was irrigated with saline solution to remove any debris or blood.

## Discussion

Contraception via intrauterine devices is one of the most widely used methods worldwide. However, their side effects, complications, and contraindications must be known to optimize performance [3].

Some authors have identified risk factors associated with uterine perforation after intrauterine device insertion, such as insertion within 0 to 3 months postpartum, and between 3 to 6 months postpartum. Increased parity decreased the risk, while an increased number of abortions increased the risk [4]. Other factors, such as the type of IUD inserted, uterine size and position, insertion timing, and clinician experience, are associated with perforation. In patients with congenital malformations or previous surgeries, uterine perforation can occur during insertion. About 15% of perforations result in complications in adjacent organs, mainly in the intestine [5]. Complete migration of the IUD into the abdominal cavity appears to result from iatrogenic uterine perforation, whether partial or complete, occurring at the time of insertion, often evidenced by initial reported pain [6] as it was reported in the second case.

These patients may present with vaginal bleeding, pelvic pain or remain asymptomatic, with the only indicator being the absence of the IUD string at the external OS of the cervix. The symptomatology of a migrating IUD is specific to its location, with atypical symptoms such as cyclical rectal bleeding, tenesmus, false needs, or associated rectal abscess, followed by a thorough clinical examination including rectal and vaginal examination, should raise suspicion of rectal localization, as reported in our first case. Ultrasound and plain abdominal X-ray can usually visualize the position of the migrating IUD. A CT scan may be necessary if previous examinations do not reveal the IUD's position [7]. It is important to note that, during pelvic ultrasound imaging, only the copper-containing IUD is visible, whereas the levonorgestrel-releasing IUD remains undetectable due to its barium-sulfate composition. Conversely, both types of IUDs are visible on plain radiography and computed tomography (CT) scans [8].

The literature has recommended immediate removal of the intrauterine device (IUD), as displacement of the IUD into the peritoneal cavity can induce the formation of peritoneal or omental adhesions, volvulus, utero-cutaneous fistulae, and intestinal perforations, leading to significant morbidity [9].

Although questions persist regarding the imperative necessity of removing an ectopic intrauterine device (IUD), conservative management may be beneficial in some circumstances, especially for asymptomatic patients [10], contrary to the present report which supports existing recommendations advocating for removal of ectopically positioned intrauterine devices (IUDs) whenever feasible [11]. According to the literature, removal of a migrating IUD is usually performed laparoscopically [9], demonstrating a documented success rate ranging from 44% to 100% [12,13].

In case of rectal perforation, the therapeutic approach aims to determine the extent of rectal wall involvement; limiting contamination of the peritoneal cavity implies removal of the intrauterine device by rectoscopy in cases of perforation with rectal mucosal involvement. In cases without mucosal involvement, removal could be performed by laparoscopy. A methylene blue test will complete the IUD removal, and in case of positivity, a transparietal suture will then be performed laparoscopically [7].

## Conclusion

During routine follow-up of patients, the observation of absent intrauterine device (IUD) strings should systematically raise suspicions of uterine perforation. This finding warrants immediate removal of the IUD under a multidisciplinary team approach to avoid serious complications.

## Patient consent

The patients gave their informed consent for this case report to be published.

## Author contributions

**Hanae Benchaou:** writing, data extraction, revision. **Ayoub Aouragh:** data extraction. **Dr Youssef El Aloua:** data extraction. **Dr Marouane Boukroute:** writing, data extraction, submission., **Abdelmajide Regragui:** iconography. **Zaineb chatbi:** writing., **Ibtissam Bellajdel:** revision. **Hafsa Taheri:** supervision of writing. **Hanane Saadi:** supervision of revision. **Ahmed Mimouni:** supervision of the work, article idea.
